# Genomic Profiling Identified Novel Prognostic Biomarkers in Chinese Midline Glioma Patients

**DOI:** 10.3389/fonc.2020.607429

**Published:** 2021-03-03

**Authors:** Hainan Li, Changguo Shan, Shengnan Wu, Baijie Cheng, Chongzu Fan, Linbo Cai, Yedan Chen, Yuqian Shi, Kaihua Liu, Yang Shao, Dan Zhu, Zhi Li

**Affiliations:** ^1^ Department of Pathology, Guangdong Sanjiu Brain Hospital, Guangzhou, China; ^2^ Medical Department, Nanjing Geneseeq Technology Inc., Nanjing, China; ^3^ Department of Pathology, Guangdong Provincial People’s Hospital, Guangzhou, China

**Keywords:** midline glioma, PI3K-AKT pathway, cell cycle pathway, prognostic genetic markers, next-generation sequencing

## Abstract

**Background:**

Molecular characteristics are essential for the classification and grading of gliomas. However, diagnostic classification of midline glioma is still debatable and substantial molecular and clinical heterogeneity within each subgroup suggested that they should be further stratified. Here, we studied the mutation landscape of Chinese midline glioma patients in hope to provide new insights for glioma prognosis and treatment.

**Methods:**

Tissue samples from 112 midline glioma patients underwent next-generation sequencing targeting 425 cancer-relevant genes. Gene mutations and copy number variations were investigated for their somatic interactions and prognostic effect using overall survival data. Pathway-based survival analysis was performed for ten canonical oncogenic pathways.

**Results:**

We identified several currently established diagnostic and prognostic biomarkers of glioma, including *TP53* (33%), *EGFR* (26%), *TERT* (24%), *PTEN* (21%), *PIK3CA* (14%)*, ATRX* (14%), *BRAF* (13%), and *IDH1/2* (6%). Among all genetic aberrations with more than 5% occurrence rate, six mutations and three copy number gains were greatly associated with poor overall survival (univariate, P < 0.1). Of these, *TERT* mutations (hazard ratio [HR], 3.00; 95% confidence interval [CI], 1.37–6.61; P = 0.01) and *PIK3CA* mutations (HR, 2.04; 95% CI, 1.08–3.84; P = 0.02) remained significant in multivariate analyses. Additionally, we have also identified a novel *MCL1* amplification (found in 31% patients) as a potential independent biomarker for glioma (multivariate HR, 2.78; 95% CI, 1.53–5.08; P < 0.001), which was seldom reported in public databases. Pathway analyses revealed significantly worse prognosis with abnormal PI3K (HR, 1.81; 95% CI, 1.12–2.95; P = 0.01) and cell cycle pathways (HR, 1.97; 95% CI, 1.15–3.37; P = 0.01), both of which stayed meaningful after multivariate adjustment.

**Conclusions:**

In this study, we discovered shorter survival in midline glioma patients with PIK3CA and TERT mutations and with abnormal PI3K and cell cycle pathways. We also revealed a novel prognostic marker, *MCL1* amplification that collectively provided new insights and opportunities in understanding and treating midline gliomas.

## Introduction

Midline glioma is not a single disease but contains multiple histological and molecular subtypes. As they are situated along the pons in the brainstem, thalamus, cerebellum, or spinal cord, which are vital for regulating basic life functions, treatment and management of these gliomas are extremely crucial (1). In the recent decade, growing evidence of molecular biology has revolutionized the classification of glioma, where molecular features make the judgment call for any discordancy between histological and molecular classifications (1–3). Based on two hallmarks of glioma, mutation of *IDH1* and *IDH2* genes, and codeletion of chromosome arms 1p/19q, glioma is primarily distinguished into five principle molecular subtypes. However, the classification of midline glioma remained controversial and the intertwining histology and molecular classifications have brought great clinical challenges. In the 2016 WHO Guidelines and the 2018 cIMPACT-NOW update, diffused midline glioma with H3 K27M-mutant was introduced as a separate Grade IV entity, predominantly describing an astrocytic differentiation of glioma of different age groups with adverse overall prognosis (1, 4). This reserved diagnostic group has left lacuna for grading H3 K27-wildtype diffuse midline gliomas, lower-grade midline-crossing tumors, and midline gliomas of other histological and morphological categories, including pilocytic astrocytoma and ganglioglioma with various survival outcomes (5–8). Meanwhile, substantial molecular heterogeneity and non-universal prognosis within each subgroup also implied the necessity of further stratification (9–11).

*H3 K27M* is found in approximately 80% of pediatric diffuse intrinsic pontine gliomas (DIPG), as well as some adult midline glioma and pediatric ependymoma at a lower occurrence (9, 12–17). Molecular analyses usually found mutually exclusive distribution of H3 K27M with the characteristic *IDH* mutations. In addition to H3 K27M, *ATRX, TP53, NF1, FGFR1, PDGFRA*, *PTPN11*, and *BRAF* alterations have been found in various pediatric and adult midline glioma subtypes, while 1p/19q co-deletion were seldom reported (8, 12, 14, 18, 19). Many of these frequently observed genes are key members of PI3K/mTOR and RAS-MAPK pathways that could potentially provide novel therapeutic options for these otherwise devastating diseases (20–24). Nevertheless, the prognostic significance of genetic aberrations and oncogenic pathways was only weakly addressed for midline glioma with or without H3 K27M mutation (20) and comprehensive molecular characterization is still lacking.

In this study, we aim to explore the genomic makeup of midline glioma patients and to fill the gaps in the prognostic heterogeneity through comprehensive genetic and oncogenic pathways analyses. As mainstay treatment for tumors in the central nervous system remained to be surgery resection, radiotherapy, and chemotherapy, we also hope to provide fresh insights into future molecular stratification in midline glioma.

## Methods

### Patients and Tumor Specimen

Archived formalin-fixed and paraffin-embedded (FFPE) blocks of tumor tissues from 112 glioma patients were obtained from the Department of Pathology at Guangdong Sanjiu Brain Hospital. Clinical characteristics and overall survival data of patients were evaluated and obtained from March 2012 to September 2019. The study was approved by the research ethics board at the hospital. All patients provided written informed consent for participating in the study.

### DNA Extraction and Sequencing Library Preparation

FFPE sections of tumor samples were sent to the CLIA/CAP-accredited central laboratory at Nanjing Geneseeq Technology Inc. (China, Nanjing) for genomic DNA extraction and hybridization capture-based targeted NGS of 425 cancer-relevant genes. Briefly, five to eight 10 µm FFPE sections were first de-paraffinized with xylene and then used for genomic DNA (gDNA) extraction by QIAamp DNA FFPE Tissue Kit (Qiagen) following the manufacturer’s instructions. The extracted gDNA samples were quantified on Qubit 3.0 fluorometer (Thermo Fisher Scientific) and its purity was measured on Nanodrop 2000 (Thermo Fisher Scientific). Purified gDNA was fragmented to a size of approximately 350 bp using a Covaris M220 sonication system (Covaris) and then purified by size selection with Agencourt AMPure XP beads (Beckman Coulter).

DNA libraries were prepared from purified gDNA with KAPA hyper library preparation kit (KAPA Biosystems) according to the manufacturer’s protocol. Libraries were then subjected to PCR amplification and purification with Agencourt AMPure XP beads before targeted enrichment.

Libraries with different sample indices were first pooled together to a total DNA amount of 2 µg and then enriched with IDT xGen Lockdown Reagents and a customized enrichment panel (Integrated DNA Technologies). The captured library was further amplified with Illumina p5 (5’ AAT GAT ACG GCG ACC ACC GA 3’) and p7 (5’ CAA GCA GAA GAC GGC ATA CGA GAT 3’) primers in KAPA Hifi HotStart ReadyMix (KAPA Biosystems, Wilmington, MA) and purified with Agencourt AMPure XP beads. Sequencing libraries were quantified by qPCR with KAPA Library Quantification kit (KAPA Biosystems), and its size distribution was examined on Bioanalyzer 2100 (Agilent Technologies). The final libraries were sequenced on Illumina Hiseq 4000 platform for 150 bp paired-end sequencing according to the manufacturer’s instructions.

### Variant Filtering and Mutation Calling

Raw sequencing data were analyzed by a validated automation pipeline. In brief, bcl2fastq was used to demultiplex raw data and trimmomatic was used to trim adapters and remove low quality reads (quality reading below 20) or N bases from FASTQ files. Burrows-Wheeler Aligner (BWA) was then used to align clean paired-end reads to the reference human genome (hs37d5). PCR deduplication was performed using Picard and indel realignment and base quality score recalibration was performed using Genome Analysis Toolkit (GATK 3.4.0) (25). Cross sample contamination was estimated using ContEst (Broad Institute) by evaluating the likelihood of detecting alternate alleles of SNPs reported in the 1000g database. The resulted mutation lists were filtered through an internally collected list of recurrent sequencing errors on the same sequencing platform. Somatic SNV and insertion/deletions (INDELs) were called using Vardict (V 1.5.4). SNVs and INDELs were further filtered using previously reported criteria. 1) for mutations with more than 20 recurrences in COSMIC, minimum variant allele frequency (VAF) = 0.01 with at least 3 minimum variant supporting reads; 2) for others, minimum VAF = 0.02 with at least 5 minimum variant supporting reads; in addition, all variants also need to meet the standards of minimum read depth = 20, minimum base quality = 25, variant supporting reads mapped to both strands, and strand bias no greater than 10%. Final mutations were annotated using vcf2maf. H3K27M mutants were detected with high specificity and sensitivity *via* immunohistochemistry using H3K27M-specific antibodies (ABE419, EMD Millipore, Billerica, MA, 1:1,000 dilution). The H3 K27M immunostaining was run in a Ventana BenchMark XT immunostainer (Ventana Medical Systems, Tucson, AZ, USA) as previously reported (26–28).

### Copy-Number Variants Analysis

Copy number (CN) analysis was performed using FACETS (Ver 0.5.13). Somatic CN alteration events were assigned based on sample-ploidy values calculated in the FACETS algorithm (29). Chromosome arm-level CN gain (>sample average ploidy +1) was defined if segments of amplification and deep amplification events account for more than 60% of total segments for the corresponding chromosome arm. Similarly, arm-level CN loss (<sample average ploidy -1) was identified if segments of deletion and deep deletion events account for more than 60% of total segments for the given chromosome.

### Pathway Analysis and Independent Validation Dataset

For pathway analysis, sets of genetic aberrations were included according to the ten previously defined canonical oncogenic signaling pathways (30). Patients harboring one or more mutated pathway member genes were considered to be positive. To validate results from pathway analysis, sequencing data from a Memorial Sloan Kettering Cancer Center cohort that contains 923 glioma patients were obtained from cbioportal (31) and the same gene inclusion criteria were applied to assign patients. *MCL1* gene expression data of 510 low-grade glioma patients were downloaded from OncoLnc.org (http://www.oncolnc.org/) (32), and survival rates were compared between high and low expression groups divided by the median RNA expression level.

### Statistical Analysis

Overall survival was defined as the time between surgery and death or last follow-up time. The association of clinical features and biomarkers with overall survival was assessed using Cox proportional hazard model. Multivariate analyses included genetic biomarkers and clinical factors with univariate P ≤ 0.1. Kaplan-Meier estimates of overall survival were illustrated and statistically compared using the log-rank test. Two-sided P < 0.5 was considered significant. All statistical analyses were done in R (3.5.0).

## Results

### Patient Characteristics and Clinical Factors

A total of 112 glioma patients were enrolled in this retrospective study, with a median age of 31 (ranging from 1 to 71 years old) and comprising 61% of male patients (**Table S1**). Histologically, 76 were diagnosed with diffused glioma (68%), 15 with neuronal and mixed neuronal-glial tumors (13%), and nine with ependymoma (8%). All tumors were in the midline location, including the brainstem, thalamus, hypothalamus, basal ganglia, lateral ventricle, fourth ventricle, and the pineal gland. According to the WHO grading scheme (1), 11 of these patients were classified as WHO grade I glioma (10%), 45 as grade II glioma (40%), 27, and 29 patients with grade III and grade IV gliomas, respectively. All patients underwent surgical resection, in which more than half experienced gross total resection. The majority of patients (76%) received postoperative radiotherapy, and 60% of all patients received adjuvant chemotherapy. Median overall survival among this cohort was 17.1 months with a median follow-up of 20.5 months. Of all clinical factors, WHO grading, the extent of surgical resection and whether radiotherapy was administered were greatly associated with survival and were included in subsequent multivariate analyses (**Table S1**).

### Identification of Prognostic Biomarkers

**Figure 1:**
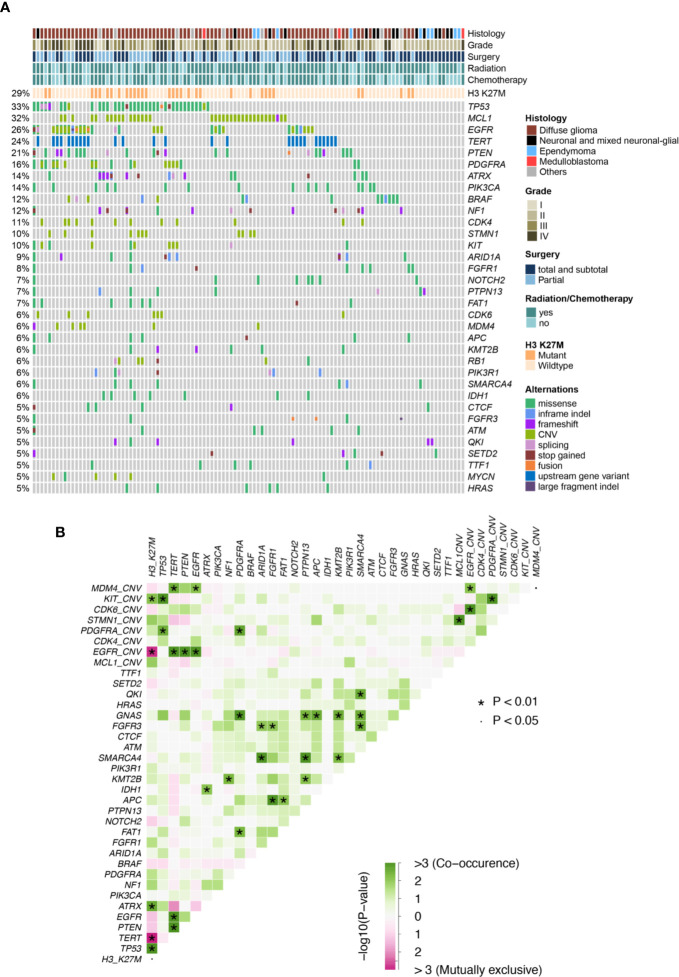
**(A)** Mutation plot of glioma patients. **(B)** Somatic interactions of midline glioma patients. Significant exclusive or co- occurrent genes were evaluated with Fisher’s exact test.

In this cohort, H3 K27M was observed in 32 patients (29%), majority of which were diffuse glioma located at the brainstem (12/32) or thalamus (11/32). Although the majority of H3 K27M-mutant tumors were diffuse glioma, we also found 1 neural-glial tumor and a few of undefined histological subtypes, with 14 pediatric and adolescent patients and 18 adults. The core glioma biomarkers, *IDH1/2* were found at a lower prevalence (8/112, 7%) and the rest 104 patients had *IDH* wild-type tumors, while no 1p/19q deletions were identified. Other previously reported midline glioma biomarkers were observed at various frequencies, including *TP53* (35/112,31%), *TERT* promoter mutations (27/112, 24%), *PTEN* (23/112, 21%), *PDGFRA* (18/112, 16%; recurrent point mutations at E227K, N659K, A810T, D842V/Y, and CN gain), *ATRX* (16/112, 14%), *PIK3CA* (16/112, 14%; at R81Q, R93W, R108H, E542K, E545K, H1047R, H1065Y), *BRAF* (14/112, 12%; primarily at V600E), *NF1* (14/112, 12%; mainly frameshift and nonsense mutations) and *FGFR1* (9/112, 8%; at G348E, N546K, V561L/M, and K656E) (**Figure 1A**, **Figure S1**, and **Table S2**). Of all the top altered genes in this cohort, we also noticed a high prevalence of *MCL1* CN gain (35/112, 31%). Next, we examined somatic interactions among these molecular events. H3 K37M was often accompanied by mutations of *ATRX* and *TP53*, and CN gain of *KIT*, while being mutually exclusive with *EGFR* and *TERT*. In contrast, *TERT* was frequently found concurrently with *PTEN* mutations, gain of *MDM4*, and alterations of *EGFR*, but not with *ATRX* as expected. Gain of *MCL1* was found to co-occurr with gain of *STMN1* (**Figure 1B**).

Only genes altered in more than 5% of patients (minimum 6 patients) were considered in the univariate analysis for their prognostic value. Clinical and genetic variables with P values ≤ 0.1 were considered for multivariate analysis, including WHO grading, the extent of resection, radiotherapy, H3 K27M, mutations of *TP53, TERT, EGFR, PIK3CA, PIK3R1*, and *APC*, as well as CN gain of *MCL1, EGFR*, and *CDK6* (**Table 1**). After adjusting for the above factors in the multivariate analysis, significantly poor overall survival was found to associate with *TERT* promoter mutations (HR, 3.00 [95% CI, 1.37–6.61]; P = 0.01), *PIK3CA* mutations (HR, 3.01 [95% CI, 1.38–6.54]; P = 0.01) and CN gain of *MCL1* (HR, 2.78 [95% CI, 1.53–5.08]; P < 0.001). In addition, marginal significance was observed in patients with CN gain of *EGFR* (HR, 2.30[95% CI, 0.97–5.48]; P = 0.06) and *APC* mutations (HR, 0.23 [95% CI, 0.05–1.05]; P = 0.06) after multivariate adjustments but predicted opposite prognostic outcomes.

**Table 1:** Prognostic significance of genetic mutations and copy number variations by univariate and multivariate cox proportional hazard model.

Variable	Population (%)	Univariate		Multivariate	
		HR (95% CI)	P value	HR (95% CI)	P value
**WHO Grade**	–	–	<0.001	–	0.09
**Surgical resection**	–	–	0.04	–	0.02
**Radiotherapy**	85(76%)	0.36 (0.21–0.63)	<0.001	0.24 (0.13–0.46)	<0.001
**H3 K27M**	32(29%)	1.73 (1.06–2.83)	0.03	1.49 (0.67–3.28)	0.33
***TP53***	35(31%)	1.52 (0.93–2.5)	0.09	1.18 (0.63–2.22)	0.6
***TERT***	27(24%)	2.17 (1.28–3.67)	0.003	3 (1.37–6.61)	0.01
***EGFR***	19(17%)	1.75 (0.98–3.13)	0.05	0.87 (0.4–1.89)	0.73
***PIK3CA***	16(14%)	2.04 (1.08–3.84)	0.02	3.01 (1.38–6.54)	0.01
***PIK3R1***	7(6%)	2.23 (0.95–5.23)	0.06	1.23 (0.46–3.32)	0.68
***APC***	7(6%)	0.30 (0.07–1.25)	0.08	0.23 (0.05–1.05)	0.06
***MCL1 CNV***	35(31%)	1.81 (1.11–2.95)	0.02	2.78 (1.53–5.08)	<0.001
***EGFR* CNV**	19(17%)	3.78 (2.09–6.85)	<0.001	2.3 (0.97–5.48)	0.06
***CDK6* CNV**	7(6%)	2.69 (1.15–6.3)	0.02	2.01 (0.63–6.43)	0.24

**Figure 2:**
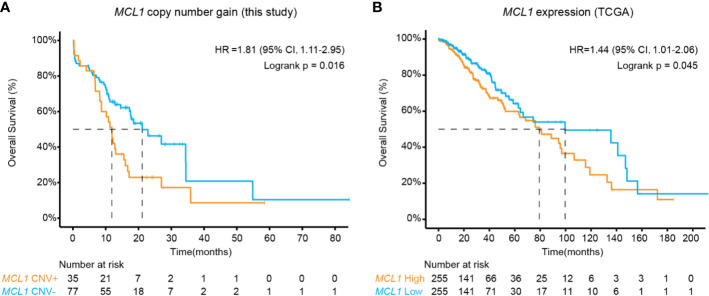
Kaplan-Meier estimates compared overall survival of patients **(A)** with or without *MCL1* copy number gain in this study and **(B)** of high and low *MCL1* expression in the TCGA cohort. P values were derived from the logrank test.

The above results revealed *MCL1* CN gain as an interesting independent biomarker of poor prognosis in midline glioma (**Figure 2A**). As the occurrence of *MCL1* CN gain was relatively low in public datasets, we further validated this finding using RNA expression data in 510 TCGA low-grade glioma patients. High *MCL1* expression was also associated with significantly worse overall outcome compared with patients with low *MCL1* expression (HR, 1.44 [95% CI, 1.01–2.06]; P = 0.045) (**Figure 2B**).

### Mutations in the Oncogenic Signaling Pathways

*PIK3CA* is an emerging biomarker in glioma as reported by several previous studies (33, 34). Our data above again supported *PIK3CA*’s role to independently predict poor prognosis in the midline cohort (**Table 1**). Because previous conclusions on the PI3K pathway in glioma were primarily restricted to the scope of PI3K kinases, PTEN, and mTOR mutations, we sought to elaborate on other PI3K pathway genes. Meanwhile, we also explored the other nine canonical oncogenic signaling pathways to evaluate whether altered pathway genes could collectively influence prognosis.

**Figure 3:**
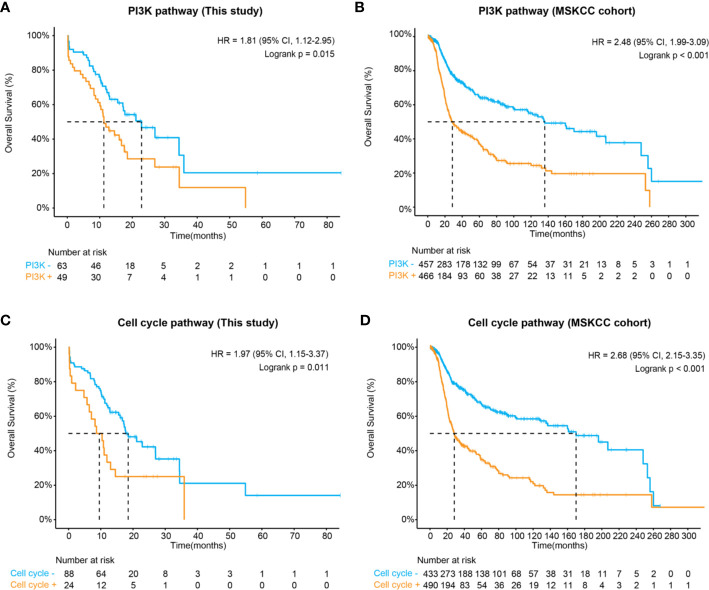
Kaplan-Meier estimates compared overall survival of patients with and without **(A)** altered PI3K pathway in this study, **(B)** altered PI3K pathway in the MSKCC cohort, **(C)** altered cell cycle pathways in this study, and **(D)** altered cell cycle pathway in the MSKCC cohort.

In concert with previous descriptions of CNS tumors (30), we have shown that the RTK/RAS (75%), PI3K (44%), TP53 (43%), cell cycle (21%), and NOTCH (21%) pathways were frequently altered in midline glioma. Of these, patients harboring at least one of the PI3K pathway mutations, including *PIK3CA, PTEN, TSC1*, *TSC2, AKT1, AKT2, AKT3, INPP4B, MTOR, PIK3R2, RICTOR*, and *RPTOR*, were significantly associated with inferior survival comparing to those without these mutations (HR, 1.81 [95% CI, 1.12–2.95]; P = 0.01) (**Figure 3A** and **Figure S2**). Importantly, a similar difference in survival was seen after considering potential bias from other clinical and genetic factors (multivariate HR, 1.85 [95% CI, 1.07–3.18]; P = 0.03) (**Table S3**). We further validated this finding in an MSKCC glioma cohort, in which 51% of patients carried one or more mutations in the PI3K pathway, comparable to our study. Again, PI3K pathway mutations were associated with shorter overall survival (HR, 2.48 [95% CI, 1.99–3.09]; P < 0.001) (**Figure 3B**).

Apart from the PI3K pathway, our data also demonstrated that the altered cell cycle pathway was significantly associated with poor prognosis (HR, 1.97 [95% CI, 1.15–3.37]; P = 0.011) (**Figure 3C**), which could be considered as an independent biomarker (multivariate HR, 0.29 [95% CI, 0.15–0.53]; P = 0.05) (**Table S3**). In the MSKCC cohort, although a higher frequency of cell cycle pathway mutations was observed (53% of patients), patients carrying these mutations also showed remarkably inferior overall survival (HR, 2.68 [95% CI, 2.15–3.68]; P < 0.001) (**Figure 3D**). Additionally, both the RTK/RAS and p53 pathways demonstrated marginal significance associated with poor prognosis in our cohort (**Table S3**). In the MSKCC cohort, the RTK/RAS pathway remained to be a significant biomarker for poor prognosis. However, similar results were not observed for the p53 pathway (**Table S4**), probably due to heterogeneity between cohorts, which might need further exploration.

## Discussion

The 2016 WHO Classification has marked the start of the molecular classification era of glioma. For midline gliomas, multiple histological and molecular subtypes have led to emerging debates in defining diagnostic entities, while the current guideline covered only one particular subtype. This unmet clinical need calls for the exploration of additional biomarkers to help stratify and clarify. In this study, we examined 425 cancer-related genes and evaluated how genetic alterations and abnormal oncogenic pathways correlated with survival outcomes in midline glioma patients. In this cohort, H3 K27M was associated with poor survival by univariate, but not multivariate analysis, probably masked by other clinical and genetic factors. Prognostication by H3 K27M could be affected by histology of the tumor. For example, in diffuse gliomas, H3 K27M was found to be a negative prognostic factor in diffuse glioma while in circumscribed and non-diffuse subtypes, it was not associated with prognosis (12, 35–37). We also found a mutually exclusive pattern of H3 K27M and *TERT*. In line with this, we identified that *TERT* promoter, *PIK3CA*, and CN gain of *EGFR* were of great prognostic significance for midline glioma despite histological subtypes. *TERT* promoter mutations and *ATRX* alterations have been described across glioma subtypes in a mutually exclusive pattern (10). In *IDH-*wildtype low-grade tumors, up to 60% of patients are *TERT* mutated, while only approximately 6% of *IDH*-mutant oligodendrogliomas are positive for *TERT*. *TERT-*mutant tumors are less responsive to surgical resection with poor survival outcomes similar to that of malignant grade IV glioblastoma across glioma subtypes, implying its prognostic role in other midline gliomas (38). Other frequently altered genes, such as *FGFR1*, *PDGFRA*, were also discovered in previous reports of DIPG and adult midline gliomas with shared recurrent mutation sites (39–42). Here, we also revealed a few novel sites including *PDGFRA* A810T and *FGFR1* V561L/M, both located within their tyrosine kinase domains.

Our comprehensive pathway analysis further elucidated that mutations in the PI3K as well as the cell cycle pathways also conferred poor patient survival. The PI3K pathway is one of the most altered pathways in *IDH*-wildtype gliomas, in consequence of *PI3K* kinase mutations, loss of *PTEN* suppressor functions, or activated mTOR (43–46) and resulted in poor prognosis (33, 34) as shown by previous researches. Here, we expanded beyond these star members of the PI3K pathway and considered a collection of pathway genes with a low abundance that were often excluded in single-gene survival analyses. Among these, *TSC1* or *TSC2* characterizes rare subependymal giant-cell astrocytomas in patients with tuberous sclerosis, causing aberrant activation of mTOR (47). By compiling a broader spectrum of genes, our data revealed that patients with any aberrant PI3K pathway mutations were greatly associated with poor survival regardless of their clinical classification. The RTK/RAS pathway was found to have a prognostic impact on pediatric thalamic glioma (20). However, in this study, this pathway showed only marginal significance univariately and no effect after multivariate adjustments, probably due to differences in the number of genes considered for pathway analysis and heterogeneity among cohorts.

Interestingly, we also unraveled CN gain of *MCL1* as a potential novel biomarker for poor survival in midline glioma. *MCL1* is a BCL-2 family of anti-apoptotic genes. Focal amplification surrounding the *MCL1* chromosome region was observed in 10% of various human cancers, promoting pro-survival signals, and preventing cell death (48). The poor prognosis associated with *MCL1* amplification or overexpression has been described in a spectrum of cancers, including non-small cell lung cancer, breast cancer, esophageal squamous cell carcinoma, and acute myeloid leukemia, but not in glioma (49–52). After a thorough literature search, we noticed that the prognostic significance of *MCL1* in glioma might be neglected as many cohort studies did not specifically cover or focus on *MCL1* gene-level amplification (9, 33, 53, 54). For instance, in the MSKCC validation dataset, only 3 patients reported CN gain of *MCL1*. In contrast, *MCL1* was relatively prevalent in roughly one-third of the entire cohort in this study, including 3 neuronal-glial tumors and 2 ependymoma. As speculated, patients with gain of *MCL1* displayed significantly shorter overall survival irrespective of their clinical status, providing the first evidence of its prognostic value in glioma. This observation was also supported by the TCGA datasets that higher *MCL1* expression was markedly associated with inferior outcomes in low-grade glioma patients. *MCL1* controls cell survival signals downstream of multiple oncogenic pathways. Co-inhibition of *MCL1* with key pathway regulators induces synergistic toxicity through different mechanisms. Of note, several clues have pointed to the canonical PI3K/AKT/mTOR pathway. In *IDH*-mutant tumors, an elevated level of the oncometabolite, 2-R-2-hydroxyglutarate, stimulates mTOR signaling that makes cells susceptible to inhibition of Bcl-xL (46, 55). Another group further demonstrated that the silencing of *MCL1* compromised cell proliferation and cell cycle signals through inhibition of the PI3K/Akt pathway (56). Such synthetic lethal interaction has not yet been studied specifically in *IDH*-wildtype gliomas. Due to the mutual exclusivity between *IDH* and *PTEN* mutations, a proposed mechanism of increased Mcl-1 in these tumors is mTOR activation by the absence of negative *PTEN* regulation. Indeed, an earlier *in vitro* study examined *PTEN*-mutated glioblastoma cell lines and demonstrated that co-inhibition of PI3K and Bcl-1 significantly depleted pAKT and Mcl-1 protein levels and enhanced cell apoptosis (57). Also, depletion of Mcl-1 by mTOR inhibitors increase vulnerability to PARP inhibitors, providing additional therapeutic options for glioma (58).

A potential limitation of this study is that a relatively small sample size of mixed histological subtypes was collected for retrospective analysis. To address potential sampling bias, the prognostic significance of genetic alterations and oncogenic pathways were examined by multivariate analyses and verified with public datasets.

In conclusion, we performed comprehensive molecular profiling of midline glioma and revealed that not only the *PIK3CA* mutations alone but aberrations on the entire PI3K pathway would together predict poor prognosis for midline gliomas. We also discovered CN gain of *MCL1* as a novel prognostic biomarker of poor survival that potentially interplays with the PI3K pathway. Collectively, our findings provided new insights in understanding and stratifying midline glioma and developing potential therapeutic targets of glioma in the future.

## Data Availability Statement

The data presented in the study are deposited in the Genome Sequence Archive for Human repository, accession number HDAC000322.

## Ethics Statement

The studies involving human participants were reviewed and approved by Guangdong Sanjiu Brain Hospital. Written informed consent to participate in this study was provided by the participants’ legal guardian/next of kin.

## Author Contributions

HL, DZ, and ZL conceptualized and designed the study. CS, SW, BC, CF, and LC managed patient information and collected samples. HL, YC, YQS, and KL performed data analysis and interpretation. HL and YC wrote the manuscript. YS, DZ, and ZL revised the manuscript. The study was supervised by ZL. All authors contributed to the article and approved the submitted version.

## Funding

This study was funded by the Guangdong Natural Science Foundation (No.2017A030313779) and the Guangdong Medical Science and Technology Research Foundation (No. A2019315).

## Conflict of Interest

YC, YQS, KL, and YS are employees of Nanjing Geneseeq Technology Inc.

The remaining authors declare that the research was conducted in the absence of any commercial or financial relationships that could be construed as a potential conflict of interest.

## Supplementary Material

Supplementary Figure 1 Lollipop graphs showing the distribution of recurrent mutation sites in the frequently mutated genes across their protein functional domains.

Supplementary Figure 2 Distribution of genetic alterations in the PI3K pathway.
