# Supplementary figures and images for: Genomic Profiling Identified Novel Prognostic Biomarkers in Chinese Midline Glioma Patients
Source: Front Oncol. 2021 Mar 3;10:607429. doi: 10.3389/fonc.2020.607429 (PMC7968371; doi:10.3389/fonc.2020.607429)

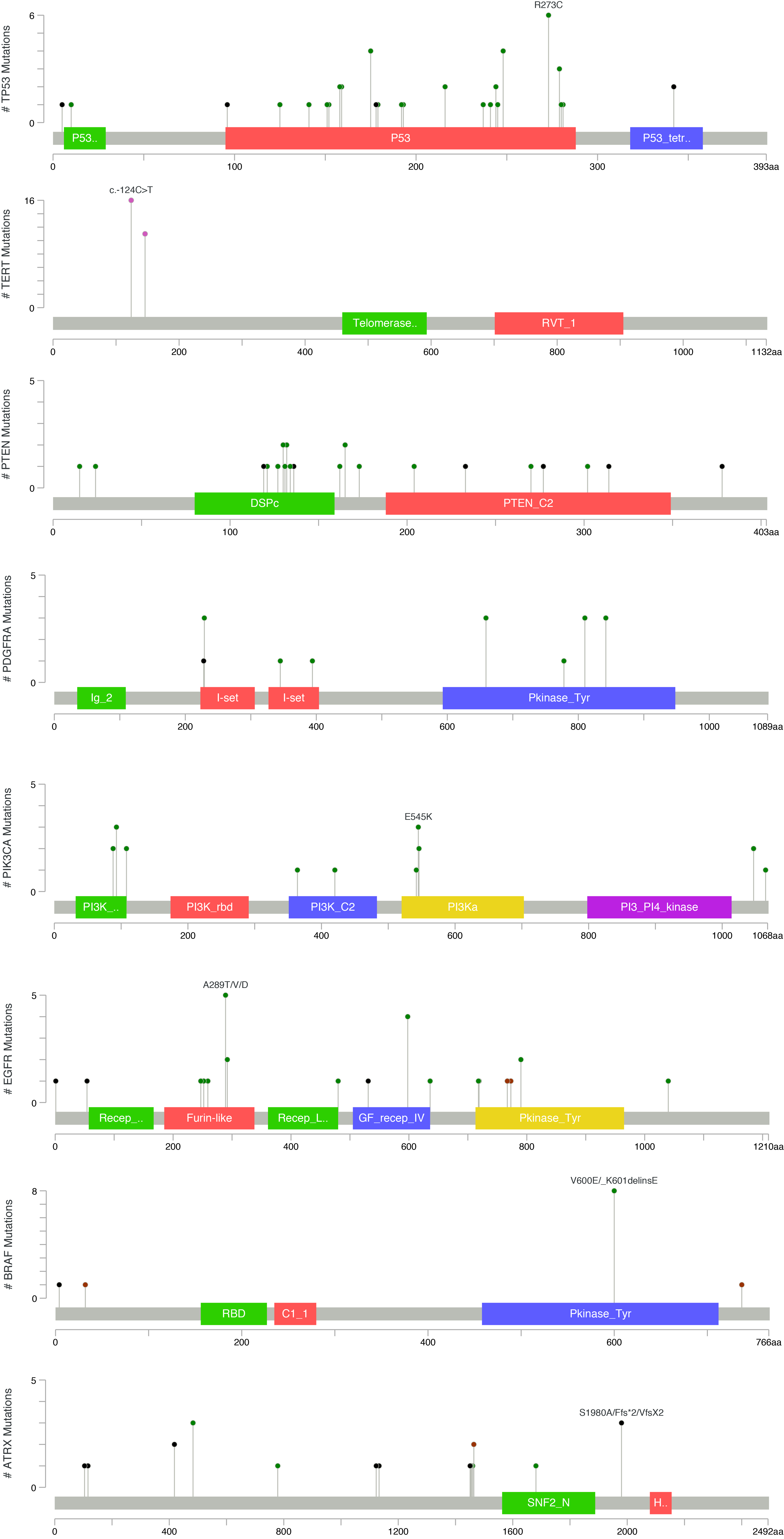

Supplement: Supplementary Figure 1 — Lollipop graphs showing the distribution of recurrent mutation sites in the frequently mutated genes across their protein functional domains. [file Image_1.jpeg]

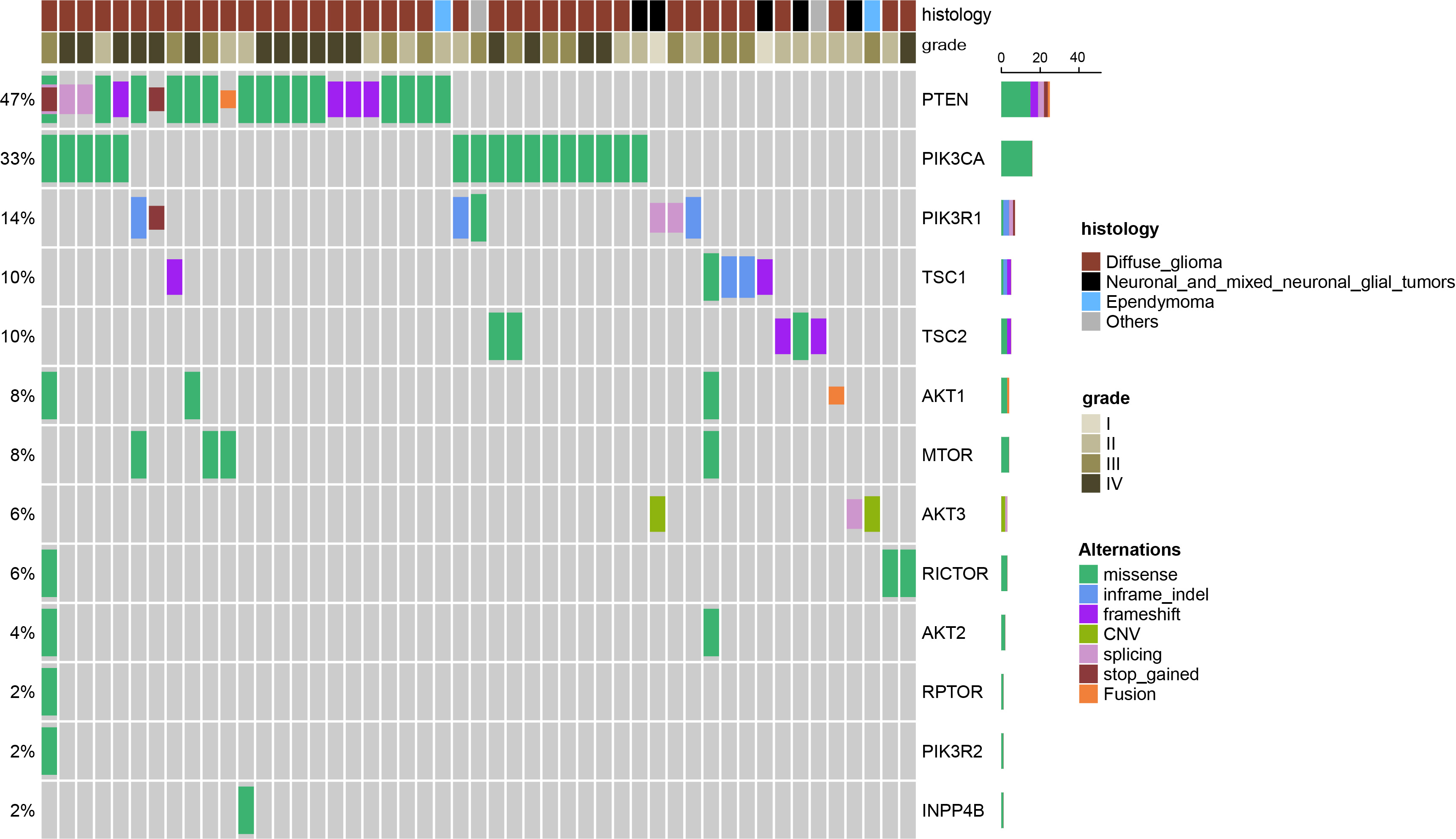

Supplement: Supplementary Figure 2 — Distribution of genetic alterations in the PI3K pathway. [file Image_2.jpeg]
